# High-resolution association mapping of number of teats in pigs reveals regions controlling vertebral development

**DOI:** 10.1186/1471-2164-15-542

**Published:** 2014-06-30

**Authors:** Naomi Duijvesteijn, Jacqueline M Veltmaat, Egbert F Knol, Barbara Harlizius

**Affiliations:** TOPIGS Research Center IPG, PO Box 43, 6640AA Beuningen, The Netherlands; Institute of Molecular and Cell Biology, A*STAR (Agency for Science, Technology and Research), 61, Biopolis Drive, Singapore, Singapore 138673

**Keywords:** Pigs, Number of teats, Vertebrae, Genome-wide association study, Somites, De-regressed breeding values

## Abstract

**Background:**

Selection pressure on the number of teats has been applied to be able to provide enough teats for the increase in litter size in pigs. Although many QTL were reported, they cover large chromosomal regions and the functional mutations and their underlying biological mechanisms have not yet been identified. To gain a better insight in the genetic architecture of the trait number of teats, we performed a genome-wide association study by genotyping 936 Large White pigs using the Illumina PorcineSNP60 Beadchip. The analysis is based on deregressed breeding values to account for the dense family structure and a Bayesian approach for estimation of the SNP effects.

**Results:**

The genome-wide association study resulted in 212 significant SNPs. In total, 39 QTL regions were defined including 170 SNPs on 13 *Sus scrofa* chromosomes (SSC) of which 5 regions on SSC7, 9, 10, 12 and 14 were highly significant. All significantly associated regions together explain 9.5% of the genetic variance where a QTL on SSC7 explains the most genetic variance (2.5%). For the five highly significant QTL regions, a search for candidate genes was performed. The most convincing candidate genes were *VRTN* and *Prox2* on SSC7, *MPP7*, *ARMC4*, and *MKX* on SSC10, and vertebrae δ-*EF1* on SSC12. All three QTL contain candidate genes which are known to be associated with vertebral development. In the new QTL regions on SSC9 and SSC14, no obvious candidate genes were identified.

**Conclusions:**

Five major QTL were found at high resolution on SSC7, 9, 10, 12, and 14 of which the QTL on SSC9 and SSC14 are the first ones to be reported on these chromosomes. The significant SNPs found in this study could be used in selection to increase number of teats in pigs, so that the increasing number of live-born piglets can be nursed by the sow. This study points to common genetic mechanisms regulating number of vertebrae and number of teats.

**Electronic supplementary material:**

The online version of this article (doi:10.1186/1471-2164-15-542) contains supplementary material, which is available to authorized users.

## Background

A favorable genetic trend for total number of piglets born has been observed in the last decade [1, 2]. Therefore, selection pressure on the number of teats has been applied in order to provide enough teats for the larger litters. Given the heritable nature of variation in number of teats in sheep as described by Bell in 1898 [3], and cases of familial supernumerary breasts (polymastia) or nipples (polythelia) in humans [4], genomic *loci* affecting teat number must exist. Indeed, the use of Best Linear Unbiased Prediction (BLUP) for estimating breeding values (EBVs) using phenotypes of both females and males, has resulted in an increase of the number of teats (data not shown) and heritability estimates are moderate with estimates between 0.2 and 0.47 [5, 6]. Besides the use of quantitative genetics to select the best sows, many studies have used genetic markers - mainly microsatellites - to identify QTL (Quantitative Trait Loci). The QTL studies on number of teats (NTE) listed in the Pig QTL database [7, 8] report QTL on all porcine chromosomes except SSC9, SSC13, SSC14, SSC18 and SSCY. Although many QTL were reported [9–18], they cover large regions and the functional mutations and underlying biological mechanisms have not yet been identified. Interestingly, some QTL for NTE seem to overlap with those for number of vertebrae [11].

Teats (or nipples) develop each as an appendage to previously formed mammary gland rudiments (MRs) during pre-natal life [19, 20]. Therefore, the number of teats correlates with the number of MRs induced and maintained at least until teat formation. The number of mammary glands varies among mammalian species, but even in humans, who normally form one pair of breasts, there are at least 6 other positions that additional breasts can randomly occupy on either side of the body [21]. Their positions range from armpit (axilla) to groin (inguen), thus span the same region where pigs form their mammary glands and teats. On both sides lateral to the ventral midline, one can draw imaginary fluent lines from both axillae to both inguenae, called mammary lines or milk lines. During embryonic life, these lines exist as histologically and molecularly distinct bands in the surface ectoderm, connecting all positions where mammary glands may form on either side of the body in any given mammalian species [22–24].

Studies on genetically engineered mice (GEMs) have revealed some insights in genetic and cellular mechanisms of mammary gland and nipple formation. Whereas wild type mice normally form five pairs of mammary glands along the mammary lines [22], modification of certain genes can alter this number [25]. For example, loss of either *Nrg3*, *Pax3*, *Gli3*, *Fgf10*, or *Hoxc6* [26–29] abolishes the formation of different, gene-specific, subsets of MRs [25]. Deletion of *e.g. p63* [30, 31] or *Tbx3* (T-box gene 3) [32] may abolish the formation of all five MRs, while reduction of Wnt signaling by means of deletion of *e.g. Lef1* (Lymphoid enhancer factor 1) [33] or *Pygo2* (Pygopus 2) allows MR induction but leads to MR regression prior to nipple formation [34]. Even if the MRs are maintained, nipple formation may not occur due to *e.g.* a lack of PTHrP (parathyroid hormone related peptide) signaling [35]. Conversely, ectodermal overexpression of the genes encoding the receptor ligands Eda-A1 (Ectodysplasin-A1) [36] or *Nrg3* [37], or suppression of the Wnt-antagonists *Lrp4* or Wise/Sostdc1, may lead to formation of one or several supernumerary mammary glands in a gene-specific pattern or region along the mammary line [38, 39]. These data provide evidence for genetic determinants for the number of mammary glands, and moreover, for the positional variation in activity of, or requirement for, these genetic determinants along the mammary line [25].

Studies carried out in the 1960’s on rabbit and mouse embryos had revealed that mammary gland formation in the surface ectoderm is initiated by factors in the dermal mesenchyme underlying the surface ectoderm [27, 40, 41]. The dermal mesenchyme is derived from the somites, which are also the precursor structures for the vertebrae and ribs. Interestingly, some of the genes mentioned above (*e.g. Gli3*, *Fgf10*, *Nrg3*, *Pax3*, *Hoxc6*)*,* are in wild type mice expressed in the somites and/or in the dermal mesenchyme. It is now known that induction of the third pair of MRs in mice, MR3, depends on Gli3-mediated *Fgf10* expression in the somites [27]. Somitic expression of Raldh2, an enzyme involved in retinoic acid synthesis, has also been associated with induction of MR3 [42].With this relationship between somitic gene expression and the number of MRs induced in mouse embryos; and knowing that somites also give rise to the vertebrae, we may expect that some QTL and candidate genes for NTE in pigs are associated with the number of vertebrae in pigs, or vertebrae development in mammalian species in general.

The availability of the Illumina PorcineSNP60 Beadchip [43] allowed us to perform a Genome-Wide Association Study (GWAS) for NTE by genotyping 936 Large White pigs. The better coverage of the whole pig genome by high-density Single Nucleotide Polymorphisms (SNPs) on these Beadchips, combined with advanced statistical methods can improve fine mapping of specific QTL as demonstrated previously for other traits [44–46]. In the current study, we based our analysis on deregressed breeding values [47] to account for the dense family structure and a Bayesian approach for estimation of the SNP effects. A total of five highly significant QTL were identified at high resolution including new regions on Sus scrofa chromosome (SSC) 9 and SSC14. Interestingly, in three of these regions we identified genes regulating vertebrae development as candidate genes determining the number of teats.

## Results

### Identification of QTL for teat number

**Figure 1:**
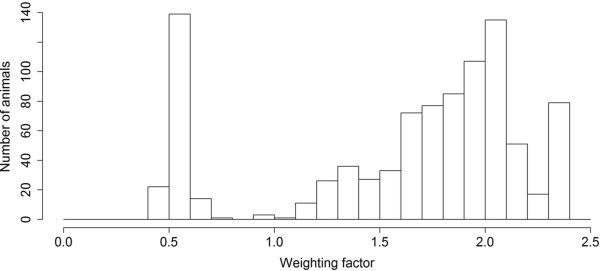
**Distribution of the weights of the breeding values calculated according to the methodology by Garrick et al.** [47]**.** The x-axis shows the weighting factors used to account for differences in phenotypic information density for each animal.

**Figure 2:**
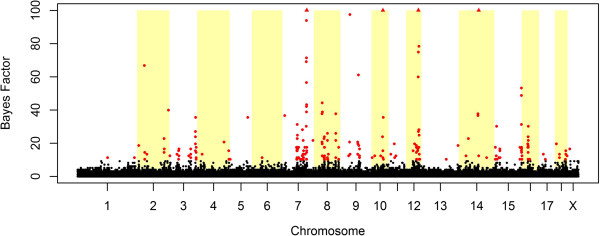
**Genome-wide association for number of teats (NTE) in 936 purebred pigs from a Large White population.** On the x-axis are the physical positions of the SNPs by chromosome. On the y-axis are Bayes Factors (BF) per SNP shown. BF <10 are black dots, BF >10 are red dots and BF > 100 are red triangles. The y-axis is cut-off at a BF of 100.

In total 949 pigs were originally genotyped, but 13 animals were removed during quality control of the data. The remaining 936 animals had an average call rate of 0.995. The mean teat number was 15.3 (SD = 0.94) with a minimum of 14 and a maximum of 19 teats. A minimum of 14 teats is used as a breeding decision, which results in a slightly skewed distribution of the phenotype in this dataset. The (deregressed) EBVs are normally distributed (results not shown). The distribution of the weighting factors calculated for NTE to account for heterogeneous variances are shown in Figure 1. The two distributions observed reflect the deviation between animals with or without offspring information. Animals with a weighting factor below 1 have no offspring where the animals above one have on average 143 offspring with records on NTE. The estimated heritability was 0.42. The GWAS resulted in 212 SNPs with a BF >10 of which 6 SNPs had a BF > 100 (Figure 2). In total 39 QTL regions were defined containing 170 SNPs on 13 chromosomes. One candidate QTL region showed an r^2^ higher than 0.7 with another candidate QTL where the distance was over 2 Mb (4 Mb respectively). These regions were combined into one region (QTL number 30 on SSC14).

**Figure 3:**
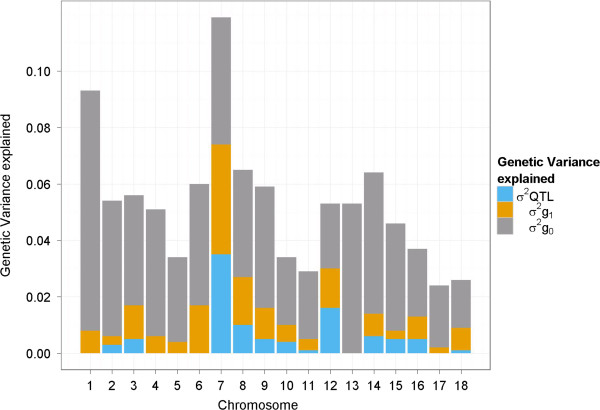
**Genetic variance explained per chromosome.** The variances explained by the defined QTL regions as in Table 1 are shown in blue (). The variances explained by SNPs when placed in the second distribution (*π*_1_) are shown in orange (). The variances explained by SNPs when placed in the null distribution (*π*_0_) are shown in grey (). The variances of the SNPs were summed per chromosome.

The 39 significantly associated regions explain 9.5% of the genetic variance with QTL number 13 on SSC7 explaining most of the genetic variance (2.5%, see Table 1). The most significant SNP within this region is ALGA0122954. The allele substitution effect of the A allele (major allele) to the G allele constitutes an additional 0.21 teat on a phenotypic level. This is an indication for overdominance because the heterozygous animals have on average 15.45 teats compared to 15.16 for AA animals and 15.37 for GG animals, respectively. The second largest QTL located on SSC12 explains more than 1% of the genetic variance, with ALGA0066876 being the SNP which explains most of the variance within this region. The allele substitution effect of the A allele (major allele) to the G allele is almost 0.09 teat indicating that the effect is completely additive (see Table 1). In total there are 14 QTL which each explain more than 0.2% of the genetic variance. In Figure 3, the explained variance per chromosome is shown. SSC7, which contains the largest QTL, is also the chromosome which explains most of the genetic variance. The second largest contributing chromosome is SSC1, but this is only due to the large number of SNPs all contributing a small variance () because SSC1 is by far the longest autosome in pigs. The total attributed SNP variance when placed in the null distribution was 69%.

**Table 1:** **Significant QTL regions per chromosome (SSC) associated with number of teats including the most significant SNPs, minor allele frequency (MAF), Bayes Factor (BF), allele substitution effect, the genetic variance explained by the region and the number of genes (Ensembl Gene IDs) found within the region**

QTL number	SSC	Region ^1^	Most sign. SNP	MAF	BF	Allele subst. effect ^2^	% Gen. var. explained region ^3^	Found genes
**1**	2	32.99-34.51	ALGA0012906	0.27	66.7	−0.143	0.17	1
**2**	2	140.44-140.93	ISU10000081	0.28	22.7	−0.17	0.11	12
**3**	3	39.13-39.28	MARC0000538	0.35	15.4	−0.079	0.04	1
**4**	3	44.55-45.82	ASGA0014296	0.29	16.4	−0.067	0.06	22
**5**	3	113.1-113.41	ALGA0020641	0.3	16.4	−0.068	0.08	1
**6**	3	134.97-135.69	ASGA0090006	0.23	35.5	−0.051	0.23	12
**7**	3	138.21-138.59	ALGA0115665	0.18	17.5	−0.117	0.07	0
**8**	4	139.72-140.10	H3GA0014860	0.39	15.4	−0.002	0.04	3
**9**	7	55.41-58.19	H3GA0021793	0.35	31.2	0.187	0.57	28
**10**	7	63.59-63.78	DRGA0007689	0.46	17.5	0.151	0.05	4
**11**	7	73.7-74.94	DRGA0007771	0.32	10.2	−0.155	0.04	1
**12**	7	87.05-91.15	ALGA0042950	0.35	28	0.157	0.29	7
**13**	7	102.01-105.22	ALGA0122954	0.21	210.1	0.227	2.51	56
**14**	8	33.26-35.00	ASGA0093882	0.34	44.3	0.16	0.38	11
**15**	8	43.92-45.19	ALGA0119079	0.23	22.7	−0.205	0.06	12
**16**	8	55.18-58.63	ALGA0047889	0.18	23.8	−0.198	0.24	26
**17**	8	80.00-81.42	MARC0044036	0.18	25.9	0.246	0.12	8
**18**	8	130.69-130.91	ALGA0049466	0.46	37.7	0.054	0.2	1
**19**	8	143.24-143.35	ALGA0050050	0.36	17.5	−0.073	0.05	1
**20**	9	36.15-38.36	UMB10000133	0.18	97.5	−0.158	0.24	20
**21**	9	75.48-76.83	ALGA0053672	0.39	20.6	−0.171	0.16	2
**22**	9	85.48-85.84	H3GA0027836	0.29	16.4	−0.17	0.07	2
**23**	10	52.21-53.94	DRGA0010548	0.09	122.3	0.39	0.29	10
**24**	10	56.37-56.78	ASGA0103067	0.48	35.5	0.101	0.09	2
**25**	11	25.34-26.23	ALGA0061540	0.3	19.5	0.171	0.05	7
**26**	12	42.93-44.76	ALGA0123748	0.25	18.5	−0.141	0.14	18
**27**	12	48.73-49.13	ASGA0082658	0.24	16.4	0.234	0.06	4
**28**	12	51.95-52.62	ALGA0066876	0.47	156.4	0.094	1.1	14
**29**	12	54.70-56.06	H3GA0034702	0.19	78.4	0.212	0.29	90
**30**	14	80.44-84.34	ALGA0079106	0.48	140.4	0.143	0.56	47
**31**	15	3.02-3.24	H3GA0043638	0.07	17.5	0.309	0.03	1
**32**	15	8.04-8.91	ASGA0068444	0.26	30.1	0.195	0.06	3
**33**	15	26.16-26.82	CASI0009989	0.17	16.4	0.235	0.05	1
**34**	15	147.34-147.74	ASGA0071500	0.25	17.5	0.246	0.04	8
**35**	15	153.82-154.57	M1GA0027067	0.4	53.1	−0.027	0.35	23
**36**	16	1.02-1.03	H3GA0045756	0.44	12.3	−0.034	0.04	0
**37**	16	27.60-30.67	DRGA0016028	0.34	30.1	0.035	0.43	24
**38**	18	21.22-21.27	H3GA0050517	0.47	13.3	−0.228	0.05	0
**39**	18	50.12-51.64	ASGA0080142	0.21	15.4	0.237	0.06	6

^1^The position in Mb of the significant (BF >10) left and right flanking markers.

^2^The allele substitution effect is the regression coefficient of the most significant SNP of the QTL on number of teats corrected for fixed effects (sex of the animal and farm). The minor allele is counted.

^3^The genetic variance explained by the QTL region expressed in %.

### Gene identification within QTL regions

Within the QTL regions, 489 genes (unique Ensembl Gene IDs) were mapped (see Additional file 1 for the genes). For the five highly significant QTL regions on SSC7, 9, 10, 12 and 14 with a BF near or larger than 100, a search for candidate genes was performed. On SSC7 between 102 and 105 Mb, a gene named *Vertnin (VRTN)* and encoding a potential DNA binding factor, is located at 103.45 Mb. The number-increase allele (Q) of this gene, was shown to add an additional thoracic segment to the pig compared to the wild-type (*wt*) [48]. Among the 14 genes annotated to the QTL area on SSC12 explaining more than 1% of the genetic variance, is *δ-EF1* mapping to 51.96 Mb. We consider this the most likely candidate gene, because it encodes transcriptional repressor involved in skeletal development [49]. On SSC10, three candidate genes were identified between 52.21 and 53.94 Mb. Of these, *MPP7*, is located at 53.01 Mb. It encodes a MAGUK (peripheral membrane-associated guanylate kinases) p55 subfamily member 7-like transcript, which is required for the establishment of cell polarity in the developing *Drosophila* embryo [50]. Also in cultured cells, MPP7 promotes epithelial cell polarity and tight junction formation [51]. The second candidate gene is *ARMC4* at position 53.34 Mb. *ARMC4* belongs to the *CTNNB1*-family of beta-catenins, which are well known for roles in transcriptional transduction of *Wnt* signaling and tissue integrity via cell-cell adhesion. The third candidate gene is *MKX* (Mohawk) at 53.68 Mb, a homeobox gene which again acts as an important regulator of vertebrae development. For the two other QTL regions on SSC9 between 36.15 and 38.46 Mb and on SSC14 between 80.44 and 84.34 Mb, no obvious candidate genes were identified.

## Discussion

### Strength of GWAS methodology

In the present study, a Bayesian Variable Selection approach was used to estimate SNP effects. By using a relatively stringent prior (*π*_1_ =0.001) on average 42 SNPs per run are selected to have an effect on the trait. To secure that many SNPs have been in the *π*_1_ distribution, the number of cycles run was large (500.000 respectively). The advantage is that less SNPs are given a large variance and therefore we expect a clearer distinction between SNPs with a small effect and SNPs with a larger effect [52]. Instead of a sliding window of N number of SNPs, which is often used to account for linkage disequilibrium (LD) [47], regions were defined based on distances between significant SNPs (<2 Mb) and LD for post-analyses. The number of SNPs within a region was variable between a single SNP up to 16 SNPs. The defined region was used to simultaneously estimate the explained variance of the SNPs within the QTL region.

The use of deregressed EBV’s should also give more reliable results from the GWAS resulting in a better estimation of the size of the SNP effects [53]. Use of only phenotypes in a GWAS from the animals genotyped without considering the information of offspring, parents and other family members is reducing the power of the study. Deregressed EBV’s are often used in dairy cattle breeding where daughter yield deviation (DYD) are used in GWAS, which have similar properties to deregressed EBV’s [54]. Deregressing breeding values is used to circumvent selecting SNPs which explain family relatedness rather than associated genes. This is done by removing the contribution of information from relatives.

### Reliability of identified QTL areas

In total 39 QTL regions were identified with relatively small effects, which suggest that NTE is controlled by many genes. This is in agreement with earlier performed linkage studies where across different pig populations many QTL were found on almost all chromosomes. Including the results from this study and published QTL, all chromosomes with the exception of SSC13 and the Y chromosome, carry QTL for teat number. Figure 3 also clearly shows that some QTL found in this study only have a limited contribution compared to the genetic variance explained by SNPs which were in the null distribution (*π*_0_). This helps to distinguish whether the variance explained per chromosome is expected because SNPs have a small variance, or alternatively, whether there is a large QTL contributing to the chromosomal genetic variance. The expected variance explained per chromosome is proportional to the number of SNPs on the chromosome with the assumption that no QTL is located on the chromosome, for example on SSC13. The difference between the total chromosomal genetic variance and the expected variance when SNPs are in the null distribution plus the QTL variance, can be caused by SNPs that were selected (*π*_1_) but did not reach the significance level to be assigned to a QTL region. The genetic variance explained by the defined QTL region on SSC7 might even be an underestimation due to SNPs which are in LD with the QTL region, but did not reach the significance threshold of a BF >10. In general, the explained variance by the identified QTL regions is small. Several factors could be underlying the relatively small explained variance. The size of this study is moderate in livestock and small compared to human studies [55]. A larger sample size could pick up more rare variants, additionally a larger SNP set (>500.000) will have the SNP closer to the causative mutation and will give more statistical power to the association study [55]. The trait NTE has also been under selection for at least 10 years (E.F. Knol, personal observation), which could have resulted in rapid fixation of large segregating QTL. Additionally, NTE is a categorical trait and not directly measuring the variation of the underlying physiological factors of the developmental signalling chain.

**Figure 4:**
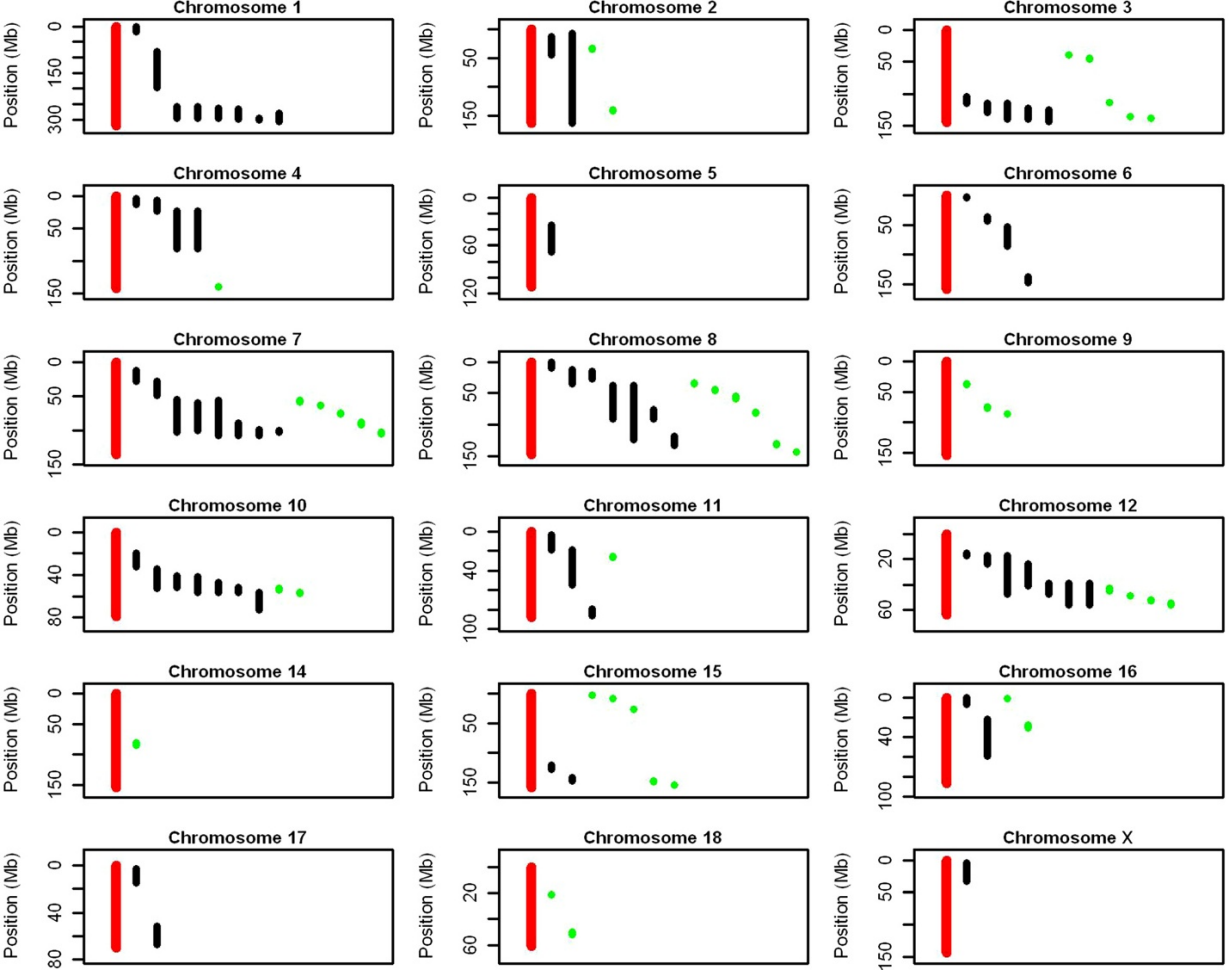
**Comparison between the QTL found in the PigQTLdb and this study per chromosome for number of teats.** Black lines are QTL reported in the PigQTLdb and the green lines indicate the QTL found in this study. The length of the bar indicates the length between the left and right flanking marker of the QTL. The red bar indicates the length of the chromosome.

Most of the QTL regions identified in this study reside within published QTL for NTE on several chromosomes (SSC2, 3, 7, 8, 10, 11, 12, 15 and 16 respectively). In Figure 4, a detailed overview of all the published QTL compared to the results from this study are shown (see Additional file 2 for the details of the studies published on NTE). Overall, the regions identified in this study are much smaller, as a result of using high-density SNP information in a GWAS instead of microsatellites as in a linkage study.

Especially on SSC7, 8, 10 and 12 some QTL are located relatively close to each other. We identify them as small individual QTL regions rather than one large QTL, because the LD (measured by r^2^) between the SNPs is less than 0.7. Besides region 30 on SSC14, the r^2^ between the closely identified QTL was always below 0.2, which suggests the QTL should be considered as independent genomic regions.

### Candidate QTL and genes

The QTL which explains the most genetic variation is located on SSC7 and has been identified in other studies [9, 11, 12, 17, 18]. While Mikawa et al. [48] reported an additive effect of this QTL on number of vertebrae, we observed a dominant or even overdominant effect on the number of teats in our population. This is in accordance with a previous study reporting that breeding for increased body length also resulted in increased teat number, leading to the speculation that the number of vertebrae is genetically linked to the number of teats [11]. Within this QTL we identified a gene named *Vertnin* (*VRTN*) at 103.45 Mb, which likely provides such a genetic link. The likeliness of *Vertnin’s* candidature is strongly supported by the previous demonstration that structural integrity of the somites, as well as somitic expression of genes such as *Gli3* and *Fgf10*, are required for the proper formation of the mammary line and glands at the axial level of thoracic/abdominal transition in mice [27]. Mechanistically, the genetic linkage between numbers of vertebrae and teats can be explained by the somites being precursors for vertebrae as well as dermal mesenchyme, the latter inducing mammary gland formation in the overlying surface ectoderm [27, 39, 40].

Another candidate gene in the region is *Prox2*, a vertebrate homolog of the *Drosophila* homeobox-containing gene prospero *Prox2* belongs to a family of transcription factors whose function has not yet been characterized in detail in mammals. In zebrafish, *Prox2* is mainly expressed in the brain and involved in eye development [56]. Notably, both *Vertnin* and *Prox2* have recently been proposed as candidates for the number of vertebrae in a White Duroc x Chinese Erhualian intercross, that also carries two different haplotypes for increased teat number [57].

The second largest QTL effect explaining more than 1% of the genetic variance is located on SSC12 between 51.95 and 52.62 Mb. Although many studies have found a QTL for NTE on SSC12 [9, 11, 12, 17, 18], only Guo et al. [12] found a large QTL interval similar to ours in an F2 cross between Large White and Chinese Meishan. Within this region, we find the δ-*EF1* gene is of particular interest because its expression in the somites [58] may suggest a role in vertebrae and mammary development in a mechanism similar to *Vertnin*, as described above.

On SSC10, we found a significant QTL in the same region as in other studies [12, 13, 15] or neighbouring a region found in other studies [16, 59]. Between 52.21 and 53.94 Mb within this region, we identified three candidate genes, namely *MPP7, ARMC4,* and *MKX*. In *Drosophila* and in cultured cells, *MPP7* promotes cell polarity and tight junction formation [50, 51]. While a role for *MPP7* and tight junctions in early mammary gland development has not yet been studied, cell polarization and tissue stratification are integral part of mammary induction and early growth, as observed in mice [23], supporting the candidature involvement of *MPP7* establishment of gland/teat number. As a member of the *β-catenin* gene family, *ARMC4* could be involved in mammary gland formation via its role in transcriptional transduction of *Wnt/β-catenin* signaling. A role for this signaling pathway in early mammary gland formation is demonstrated by compromised mammary gland formation in the presence of pathway-inhibitory mutations, and supernumerary mammary gland formation in the context of excessive Wnt signaling [33, 34, 60–63]. Alternatively, Wnt signaling is implicated downstream of *PTHrP* signaling in nipple formation, which is a process that occurs secondary to the formation of mammary rudiments [64].

Most convincing, the third gene *MKX* is in mice expressed in the condensing mesenchyme that will ultimately become the proximal ribs and vertebral bodies [65].

The two other QTL regions on SSC9 between 36.15 and 38.46 Mb and on SSC14 between 80.44 and 84.34 Mb are new and have not been reported before. Soma et al. [66] reported a QTL for number of lumbar vertebrae on SSC14 around 137.88 Mb (119 cM, marker SW761) in a Duroc purebred population, but the QTL peak is located around 55 Mb further distal of our QTL no. 30 (SSC14: 80.44-84.34 Mb).

Elongation of the back and an increased number of vertebrae in pigs as a consequence of domestication was already observed by Charles Darwin [67]. Signatures of selection have also been found for numbers of vertebrae and body length in the domestic pig [68] and for number of teats [69]. In accordance, some QTL for number of vertebrae and number of teats in pigs overlap [11]. A mutation increasing number of teats in the gene *NR6A1* (an orphan nuclear receptor) on SSC1 has been fixed in commercial breeds [70] and therefore, this QTL is not segregating in our population either. Nevertheless, selection for increased carcass length still provides variation for genetic improvement of reproductive traits such as number of teats in the sow which is in turn very relevant for the survival of piglets.

Mechanistically, the link between carcass length (number of vertebrae) and number of teats can be explained by results from studies in mice, revealing a role for the somites, *i.e.* precursor structures for vertebrae, in the induction of mammary gland development [22, 25, 27]. Although to our knowledge, a variation in number of somites (vertebrae) in mice has not been subject to study in mice, and certainly not in relationship to the number of mammary glands or nipples/teats, it is clear that altered somitic development or gene expression can alter the number of mammary glands (thus nipples/teats) in mice [22, 25, 27]. In agreement with this biological mechanism of mammary gland development, we identified in our current study several candidate genes with a known association to vertebrae development. To date, none of these genes have a reported role in mammary gland or nipple/teat development. Such studies could certainly be helpful to get closer to the causative genetic variant which can be used in breeding programs for increased NTE in pigs.

## Conclusion

Although NTE has been under selection for many generations, this study found many QTL controlling NTE. We could narrow down considerably some of the earlier published QTL regions making it easier to select candidate genes. Five major QTL were found at high resolution on SSC7, 9, 10, 12, and 14 of which the QTL on SSC9 and SSC14 are the first ones to be reported on these chromosomes. The confirmed major QTL found on SSC7 contains the candidate gene *Vertnin* which has been reported to control the number of thoracic vertebrae. Interestingly, also the two other regions on SSC12 and SSC10 contain genes that have a suspected (δ-*EF1*) or demonstrated (*MKX*) involvement in vertebrae development. The genetic relation between teat number and number of vertebrae can be explained by the somites being precursors for both vertebrae and dermal mesenchyme, while both the somites and dermal mesenchyme have been shown to contain inductive signals for mammary gland formation. All significant QTL together explain almost 10% of the genetic variance. Nevertheless, results clearly show the polygenic nature of the trait indicating the genetic complexity of the trait. The significant SNPs found in this study could be used in selection to increase NTE in pigs, so that the increasing number of live-born piglets can be nursed by the sow.

## Methods

### Animals

This study was conducted strictly in line with the regulations of the Dutch law on the protection of animals (Gezondheids- en welzijnswet voor dieren). Animal Care and Use Committee approval was not obtained for this study, because the data were obtained from an existing database. Phenotypic measurements of the number of teats (NTE) were obtained from 936 pigs, of which 230 were boars and 706 sows. All pigs were purebred Large White and were born between 2006 and 2009 and originated from 17 farms. The number of teats were counted at birth and recorded on both sexes. In this study, NTE was the only recorded trait and number of left and right teats and teat malformations were not considered.

The boars originated from 100 sires (29 genotyped) and 148 dams (20 genotyped). The sows originated from 164 sires (31 genotyped) and 464 dams (48 genotyped). The boars had between 0 to 3,052 offspring with phenotypic observations and the sows between 0 and 180 offspring with phenotypic observations.

### Genotyping and SNP quality

Genotyping was performed using the Illumina PorcineSNP60 Beadchip. Samples collected for DNA extraction were only used for routine diagnostic purpose of the breeding program and was strictly in line with the Dutch law on the protection of animals (Gezondheids- en welzijnswet voor dieren). DNA was extracted from blood, hair and ear punches and commercially genotyped at Service XS (Leiden, The Netherlands) or at Geneseek, Inc. (Lincoln, NE, USA). SNPs with a GenCall score <0.7, call rate <95%, minor allele frequency <0.01 and SNPs with no physical position on the pig genome (pig genome build10.2) were removed. After these quality control measures, 42,654 SNPs out of 64,232 SNPs remained for the genome-wide association (GWA).

### Statistical method for GWA analyses

The estimated breeding value (EBV) for every animal was obtained via routine genetic evaluation using MiXBLUP [71] in a multitrait model. The model for obtaining the EBV for NTE included fixed effects for herd-year-season, sex and line and an additive genetic effect (animal) as a random effect. Reliabilities per animal were extracted from the genetic evaluation and was based on the methodology of Tier and Meyer [72]. The EBV was deregressed using the methodology proposed by Garrick et al. [47].

The deregressed EBV only contains information of the animals’ own performance and of their descendants’, which was achieved by removing the parent average. The reliabilities (information sources) vary considerably between animals and therefore the deregressed EBVs have heterogeneous variances. This is resolved by weighing the residuals as in Garrick et al. [47]. Deregression of the EBV was applied to account for the dense family structure in the data and the large difference in the number of information sources to avoid double counting.

A Bayesian Variable Selection model [73] was fitted for NTE by estimating the marker effects with all SNPs simultaneously in the model:


where **y** is a n-vector of phenotypes on n animals, μ is a n-vector equal to the mean, X is a n by p matrix where p SNPs are coded as 0, 1, or 2 copies of a particular allele vector and β is a p-vector with the marker effects. A Bernoulli distribution is applied on the marker effect:


where the first distribution is referred to the null distribution and SNPs are assumed to have a small effects () and the second distribution contains SNPs assumed to have a large effect explaining a large variance () of the phenotype. The probability to be in the null distribution (*π*_0_) was set to 0.999, meaning only 1 in every 1000 SNPs will be in the second distribution which is on average 42 SNP per cycle. The term *e* is a n-vector with random residual effects assumed to be normally distributed but weighted, , where W is a diagonal matrix with elements *w*_1_, …, *w*_*n*_. The model was implemented in the program Bayz [74].

A total of 500,000 MCMC chains with a burn-in of 5,000 cycles were run and a Metropolis-Hastings sampler was applied to get good convergence which was assessed by visual inspection of the trace and using Gelman and Rubin’s convergence diagnostic based on deviance [75] using the R package CODA [76].

### Identification of associated regions

To determine which SNPs are significantly associated, a Bayes Factor was calculated for every SNP using the prior probability (*π*_0_ and *π*_1_) and the posterior probability () by calculating an odds ratio as:


A BF >10 is referred to as ‘strong’ and a value above 100 as ‘decisive’ [77].

When at least two SNPs in a region (<2 Mb) showed a BF >10, this region was defined as a candidate QTL region and for regions with a BF around or over 100, a gene search was conducted. To define a QTL for NTE in this analysis, linkage disequilibrium (LD) was taken into account. When r^2^ was >0.7 between two QTL regions, but the distance was larger than 2 Mb, the region was still combined into one common region.

The variance explained by the QTL region was calculated by simultaneously estimating the variance explained by all the regions and all the other non-significant SNPs. To get insight in the chromosomal partitioning of the genetic variance, SNPs within a QTL region (BF > 10) and SNPs on different chromosomes were placed in different groups. The sum of the variance explained per chromosome was the sum of variances of the QTL (if detected) on a chromosome plus the explained variance by the other SNPs on the chromosome. The variance expected per chromosome was calculated as the average variance explained by SNPs when placed in the null distribution (*π*_0_).

### QTL comparisons and candidate genes

All earlier reported QTL found for NTE were available at the PigQTLdb (http://www.animalgenome.org/QTLdb/pig.html). Flanking markers of the QTL were searched at the reference genome (build10.2) to find the physical position of the markers. If one of the markers was not found, a BLAST search was performed. If the physical position of markers could not be identified the closest neighboring marker according to the linkage map from MARC USDA (http://www.marc.usda.gov/genome) was mapped. When any of these markers could not be placed on the physical map, the QTL was not included in the comparison.

For gene searches, the left and right flanking markers of the defined candidate QTL regions were used. Pig genome build10.2 was used for the position of the SNPs. Gene annotation for QTL regions was performed with BIOMART software in the Ensembl Sscrofa 10.2 (http://www.ensembl.org). Ensemble Gene IDs were used to count the number of genes within the QTL regions.

## Electronic supplementary material

Additional file 1: **All 489 genes found in QTL regions as reported in Table 1.** The genes are mapped on build10.2. Ensemble gene id was given, start and end of the gene, the status of the transcript and gene and when known the gene name and function. (XLSX 45 KB)

Additional file 2: **Overview of the studies reporting QTL on number of teats.** The position of the microsatellites are given in Mb and were mapped on build10.2 [9, 18, 59, 78, 85]. (XLSX 14 KB)

## References

[1] Merks JWM, Mathur PK, Knol EF (2012). New phenotypes for new breeding goals in pigs. Animal.

[2] Neeteson-van Nieuwenhoven A-M, Knap P, Avendaño S (2013). The role of sustainable commercial pig and poultry breeding for food security. Animal Frontiers.

[3] Bell AG (1898). On the development by selection of supernumerary mammae in sheep. Science.

[4] Schmidt H (1998). Supernumerary nipples: prevalence, size, sex and side predilection -a prospective clinical study. Eur J Pediatr.

[5] Chalkias H, Rydhmer L, Lundeheim N (2013). Genetic analysis of functional and non-functional teats in a population of Yorkshirepigs. Livest Sci.

[6] McKay RM, Rahnefeld GW (1990). Heritability of teat number in swine. Can J Anim Sci.

[7] Hu ZL, Park CA, Wu XL, Reecy JM (2013). Animal QTLdb: an improved database tool for livestock animal QTL/association data dissemination in the post-genome era. Nucleic Acids Res.

[8] Hu ZL, Fritz ER, Reecy JM (2007). AnimalQTLdb: a livestock QTL database tool set for positional QTL information mining and beyond. Nucleic Acids Res.

[9] Bidanel JP, Rosendo A, Iannuccelli N, Riquet J, Gilbert H, Caritez JC, Billon Y, Amigues Y, Prunier A, Milan D (2008). Detection of quantitative trait loci for teat number and female reproductive traits in Meishan X Large White F2 pigs. Animal.

[10] Cassady JP, Johnson RK, Pomp D, Rohrer GA, Van Vleck LD, Spiegel EK, Gilson KM (2001). Identification of quantitative trait loci affecting reproduction in pigs. J Anim Sci.

[11] Ding N, Guo Y, Knorr C, Ma J, Mao H, Lan L, Xiao S, Ai H, Haley CS, Brenig B, Huang L (2009). Genome-wide QTL mapping for three traits related to teat number in a White Duroc x Erhualian pig resource population. BMC Genet.

[12] Guo YM, Lee GJ, Archibald AL, Haley CS (2008). Quantitative trait loci for production traits in pigs: a combined analysis of two Meishan x Large White populations. Anim Genet.

[13] Hirooka H, De Koning DJ, Harlizius B, Van Arendonk JA, Rattink AP, Groenen MA, Brascamp EW, Bovenhuis H (2001). A whole-genome scan for quantitative trait loci affecting teat number in pigs. J Anim Sci.

[14] Holl JW, Cassady JP, Pomp D, Johnson RK (2004). A genome scan for quantitative trait loci and imprinted regions affecting reproduction in pigs. J Anim Sci.

[15] Rodriguez C, Tomas A, Alves E, Ramirez O, Arque M, Munoz G, Barragan C, Varona L, Silio L, Amills M, Noguera JL (2005). QTL mapping for teat number in an Iberian x Meishan pig intercross. Anim Genet.

[16] Rohrer GA (2000). Identification of quantitative trait loci affecting birth characters and accumulation of backfat and weight in a Meishan-White Composite resource population. J Anim Sci.

[17] Sato S, Atsuji K, Saito N, Okitsu M, Komatsuda A, Mitsuhashi T, Nirasawa K, Hayashi T, Sugimoto Y, Kobayashi E (2006). Identification of quantitative trait loci affecting corpora lutea and number of teats in a Meishan x Duroc F2 resource population. J Anim Sci.

[18] Zhang J, Xiong Y, Zuo B, Lei M, Jiang S, Li F, Zheng R, Li J, Xu D (2007). Detection of quantitative trait loci associated with several internal organ traits and teat number trait in a pig population. J Genet Genomics.

[19] Propper AY, Howard BA, Veltmaat JM (2013). Prenatal morphogenesis of mammary glands in mouse and rabbit. J Mammary Gland Biol Neoplasia.

[20] Veltmaat JM, Mailleux AA, Thiery JP, Bellusci S (2003). Mouse embryonic mammogenesis as a model for the molecular regulation of pattern formation. Differentiation.

[21] Dixon JM (2012). ABC of Breast Diseases.

[22] Veltmaat JM, Van Veelen W, Thiery JP, Bellusci S (2004). Identification of the mammary line in mouse by Wnt10b expression. Dev Dynam.

[23] Lee MY, Racine V, Jagadpramana P, Sun L, Yu W, Du T, Spencer-Dene B, Rubin N, Le L, Ndiaye D, Bellusci S, Kratochwil K, Veltmaat JM (2011). Ectodermal Influx and Cell Hypertrophy Provide Early Growth for All Murine Mammary Rudiments, and Are Differentially Regulated among Them by Gli3. PLoS One.

[24] Propper AY (1978). Wandering epithelial cells in the rabbit embryo milk line: a preliminary scanning electron microscope study. Dev Biol.

[25] Veltmaat JM, Ramsdell AF, Sterneck E (2013). Positional Variations in Mammary Gland Development and Cancer. J Mammary Gland Biol Neoplasia.

[26] Howard B, Panchal H, McCarthy A, Ashworth A (2005). Identification of the scaramanga gene implicates Neuregulin3 in mammary gland specification. Gene Dev.

[27] Veltmaat JM, Fdr R, Le LT, Kratochwil K, Sala FG, Van Veelen W, Rice R, Spencer-Dene B, Mailleux AA, Rice DP, Thiery JP, Bellusci S (2006). Gli3-mediated somitic Fgf10 expression gradients are required for the induction and patterning of mammary epithelium along the embryonic axes. Development.

[28] Mailleux AA, Spencer-Dene B, Dillon C, Ndiaye D, Savona-Baron C, Itoh N, Kato S, Dickson C, Thiery JP, Bellusci S (2002). Role of FGF10/FGFR2b signaling during mammary gland development in the mouse embryo. Development.

[29] Garcia-Gasca A, Spyropoulos DD (2000). Differential mammary morphogenesis along the anteroposterior axis in Hoxc6 gene targeted mice. Dev Dynam.

[30] Mills AA, Zheng B, Wang XJ, Vogel H, Roop DR, Bradley A (1999). p63 is a p53 homologue required for limb and epidermal morphogenesis. Nature.

[31] Yang A, Schweitzer R, Sun D, Kaghad M, Walker N, Bronson RT, Tabin C, Sharpe A, Caput D, Crum C, McKeon F (1999). p63 is essential for regenerative proliferation in limb, craniofacial and epithelial development. Nature.

[32] Eblaghie MC, Song S, Kim J, Akita K, Tickle C, Jung H (2004). Interactions between FGF and Wnt signals and Tbx3 gene expression in mammary gland initiation in mouse embryos. J Anat.

[33] van Genderen C, Okamura RM, Farinas I, Quo RG, Parslow TG, Bruhn L, Grosschedl R (1994). Development of several organs that require inductive epithelial-mesenchymal interactions is impaired in LEF-1-deficient mice. Gene Dev.

[34] Gu B, Sun P, Yuan Y, Moraes RC, Li A, Teng A, Agrawal A, Rheaume C, Bilanchone V, Veltmaat JM, Takemaru KI, Millar SE, Lee EYHP, Lewis MT, Li B, Dai X (2009). Pygo2 expands mammary progenitor cells by facilitating histone H3 K4 methylation. J Cell Biol.

[35] Foley J, Dann P, Hong J, Cosgrove J, Dreyer B, Rimm D, Dunbar M, Philbrick W, Wysolmerski JJ (2001). Parathyroid hormone-related protein maintains mammary epithelial fate and triggers nipple skin differentiation during embryonic breast development. Development.

[36] Mustonen T, Ilmonen M, Pummila M, Kangas AT, Laurikkala J, Jaatinen R, Pispa J, Gaide O, Schneider P, Thesleff I, Mikkola ML (2004). Ectodysplasin A1 promotes placodal cell fate during early morphogenesis of ectodermal appendages. Development.

[37] Panchal H, Wansbury O, Parry S, Ashworth A, Howard B (2007). Neuregulin3 alters cell fate in the epidermis and mammary gland. BMC Dev Biol.

[38] Ahn Y, Sims C, Logue JM, Weatherbee SD, Krumlauf R (2013). Lrp4 and Wise interplay controls the formation and patterning of mammary and other skin appendage placodes by modulating Wnt signaling. Development.

[39] Närhi K, Tummers M, Ahtiainen L, Itoh N, Thesleff I, Mikkola ML (2012). Sostdc1 defines the size and number of skin appendage placodes. Dev Biol.

[40] Propper A, Gomot L (1967). Tissue interactions during organogenesis of the mammary gland in the rabbit embryo. C R Acad Sci Hebd Seances Acad Sci D.

[41] Kratochwil K (1969). Organ specificity in mesenchymal induction demonstrated in the embryonic development of the mammary gland of the mouse. Dev Biol.

[42] Cho KW, Kwon HJ, Shin JO, Lee JM, Cho SW, Tickle C, Jung HS (2012). Retinoic acid signaling and the initiation of mammary gland development. Dev Biol.

[43] Ramos AM, Crooijmans RPMA, Affara NA, Amaral AJ, Archibald AL, Beever JE, Bendixen C, Churcher C, Clark R, Dehais P, Hansen MS, Hedegaard J, Hu ZL, Kerstens HH, Law AS, Megens HJ, Milan D, Nonneman DJ, Rohrer GA, Rothschild MF, Smith TPL, Schnabel RD, Van Tassel CP, Taylor JF, Wiedmann RT, Schook LB, Groenen MAM (2009). Design of a high density SNP genotyping assay in the pig using SNPs identified and characterized by next generation sequencing technology. PLoS One.

[44] Duijvesteijn N, Knol EF, Merks JWM, Crooijmans RPMA, Groenen MAM, Bovenhuis H, Harlizius B (2010). A genome-wide association study on androstenone levels in pigs reveals a cluster of candidate genes on chromosome 6. BMC Genet.

[45] Fan B, Onteru SK, Du ZQ, Garrick DJ, Stalder KJ, Rothschild MF (2011). Genome-wide association study identifies loci for body composition and structural soundness traits in pigs. PLoS One.

[46] Onteru SK, Fan B, Nikkilä MT, Garrick DJ, Stalder KJ, Rothschild MF (2011). Whole-genome association analyses for lifetime reproductive traits in the pig. J Anim Sci.

[47] Garrick DJ, Taylor JF, Fernando RL (2009). Deregressing estimated breeding values and weighting information for genomic regression analyses. Genet Sel Evol.

[48] Mikawa S, Sato S, Nii M, Morozumi T, Yoshioka G, Imaeda N, Yamagushi T, Awata T (2011). Identification of a second gene associated with variation in vertebral number in domestic pigs. BMC Genet.

[49] Bellon E, Luyten FP, Tylzanowski P (2009). d-EF1 is a negative regulator of Ihh in the developing growth plate. J Cell Biol.

[50] Bohl J, Brimer N, Lyons C, Pol SBV (2007). The stardust family protein MPP7 forms a tripartite complex with LIN7 and DLG1 that regulates the stability and localization of DLG1 to cell junctions. J Bio Chem.

[51] Stucke VM, Timmerman E, Vandekerckhove J, Gevaert K, Hall A (2007). The MAGUK protein MPP7 binds to the polarity protein hDlg1 and facilitates epithelial tight junction formation. Mol Biol Cell.

[52] Van den Berg I, Fritz S, Boichard D (2013). QTL fine mapping with Bayes C(π): a simulation study. Genet Sel Evol.

[53] Ostersen T, Christensen OF, Henryon M, Nielsen B, Su G, Madsen P (2011). Deregressed EBV as the response variable yield more reliable genomic predictions than traditional EBV in pure-bred pigs. Genet Sel Evol.

[54] Bolormaa S, Pryce JE, Hayes BJ, Goddard ME (2010). Multivariate analysis of a genome-wide association study in dairy cattle. J Dairy Sci.

[55] Visscher PM, Brown MA, McCarthy MI, Yang J (2012). Five years of GWAS discovery. Am J Hum Genet.

[56] Pistocchi A, Bartesaghi S, Cotelli F, Del Giacco L (2008). Identification and expression pattern of zebrafish prox2 during embryonic development. Dev Dynam.

[57] Ren DR, Ren J, Ruan GF, Guo YM, Wu LH, Yang GC, Zhou LH, Li L, Zhang ZY, Huang LS (2012). Mapping and fine mapping of quantitative trait loci for the number of vertebrae in a White Duroc x Chinese Erhualian intercross resource population. Anim Genet.

[58] Ji F, Sekido R, Murai K, Kamachi Y, Kondoh H (1993). d-crystallin enhancer binding protein dEF1 is a zinc finger-homeodomain protein implicated in postgastrulation embryogenesis. Development.

[59] Dragos-Wendrich M, Moser G, Bartenschlager H, Reiner G, Geldermann H (2003). Linkage and QTL mapping for Sus scrofa chromosome 10. J Anim Breed Genet.

[60] Boras-Granic K, Chang H, Grosschedl R, Hamel PA (2006). Lef1 is required for the transition of Wnt signaling from mesenchymal to epithelial cells in the mouse embryonic mammary gland. Dev Biol.

[61] Chu EY, Hens J, Andl T, Kairo A, Yamaguchi TP, Brisken C, Glick A, Wysolmerski JJ, Millar SE (2004). Canonical WNT signaling promotes mammary placode development and is essential for initiation of mammary gland morphogenesis. Development.

[62] Lindvall C, Evans NC, Zylstra CR, Li Y, Alexander CM, Williams BO (2006). The Wnt signaling receptor Lrp5 is required for mammary ductal stem cell activity and Wnt1-induced tumorigenesis. J Biol Chem.

[63] Lindvall C, Zylstra CR, Evans N, West RA, Dykema K, Furge KA, Williams BO (2009). The Wnt co-receptor Lrp6 is required for normal mouse mammary gland development. PLoS One.

[64] Hiremath M, Dann P, Fischer J, Butterworth D, Boras-Granic K, Hens J, Van Houten J, Shi W, Wysolmerski JJ (2012). Parathyroid hormone-related protein activates Wnt signaling to specify the embryonic mammary mesenchyme. Development.

[65] Anderson DM, Arredondo J, Hahn K, Valente G, Martin JF, Wilson-Rawls J, Rawls A (2006). Mohawk is a novel homeobox gene expressed in the developing mouse embryo. Dev Dynam.

[66] Soma Y, Uemoto Y, Sato S, Shibata T, Kadowaki H, Kobayashi E, Suzuki K (2011). Genome-wide mapping and identification of new quantitative trait loci affecting meat production, meat quality, and carcass traits within a Duroc purebred population. J Anim Sci.

[67] Darwin C (1868). The Variation of Animals and Plants under Domestication.

[68] Rubin CJ, Megens HJ, Barrio AM, Maqbool K, Sayyab S, Schwochow D, Wang C, Carlborg O, Jern P, Jorgensen CB, Archibald AL, Fredholm M, Groenen MAM, Anderson L (2012). Strong signatures of selection in the domestic pig genome. Proc Natl Acad Sci U S A.

[69] Wilkinson S, Lu ZH, Megens H-J, Archibald AL, Haley C, Jackson IJ, Groenen MAM, Crooijmans RPMA, Ogden R, Wiener P (2013). Signatures of diversifying selection in European pig breeds. PLoS Genet.

[70] Mikawa S, Morozumi T, Shimanuki SI, Hayashi T, Uenishi H, Domukai M, Okumura N, Awata T (2007). Fine mapping of a swine quantitative trait locus for number of vertebrae and analysis of an orphan nuclear receptor, germ cell nuclear factor (NR6A1). Genome Res.

[71] Mulder HA, Lidauer M, Strandén I, Mäntysaari EA, Pool MH, Veerkamp RF (2012). MiXBLUP Manual.

[72] Tier B, Meyer K (2004). Approximating prediction error covariances among additive genetic effects within animals in multiple-trait and random regression models. J Anim Breed Genet.

[73] George EI, McCulloch RE (1993). Variable selection via Gibbs sampling. J Am Stat Assoc.

[74] Janss L (2011). **BayZ manual (available at****http://bayz.biz/****)**.

[75] Gelman A, Rubin DB (1992). Inference from iterative simulation using multiple sequences. Stat Sci.

[76] Plummer M, Best N, Cowles K, Vines K (2006). CODA: Convergence diagnosis and output analysis for MCMC. R news.

[77] Kass RE, Raftery AE (1995). Bayes factors. J Am Stat Assoc.

[78] King AH, Jiang Z, Gibson JP, Haley CS, Archibald AL (2003). Mapping quantitative trait loci affecting female reproductive traits on porcine chromosome 8. Biol Reprod.

[79] Wada Y, Akita T, Awata T, Furukawa T, Sugai N, Ishii K, Ito Y, Kobayashi E, Mikawa S, Yasue H, Sugai N, Inage Y, Kusumoto H, Matsumoto T, Miyake M, Murase A, Shimanuki S, Sugiyama T, Uchida Y, Yanai S (2000). Quantitative trait loci (QTL) analysis in a Meishan X Göttingen cross population. Anim Genet.

[80] Beeckmann P, Moser G, Bartenschlager H, Reiner G, Geldermann H (2003). Linkage and QTL mapping for Sus scrofa chromosome 8. J Anim Breed Genet.

[81] Lee SS, Chen Y, Moran C, Cepica S, Reiner G, Bartenschlager H, Moser G, Geldermann H (2003). Linkage and QTL mapping for Sus scrofa chromosome 2. J Anim Breed Genet.

[82] Lee SS, Chen Y, Moran C, Stratil A, Reiner G, Bartenschlager H, Moser G, Geldermann H (2003). Linkage and QTL mapping for Sus scrofa chromosome 5. J Anim Breed Genet.

[83] Cepica S, Reiner G, Bartenschlager H, Moser G, Geldermann H (2003). Linkage and QTL mapping for Sus scrofa chromosome X. J Anim Breed Genet.

[84] Beeckmann P, Schröffel J, Moser G, Bartenschlager H, Reiner G, Geldermann H (2003). Linkage and QTL mapping for Sus scrofa chromosome 1. J Anim Breed Genet.

[85] Yue G, Schröffel J, Moser G, Bartenschlager H, Reiner G, Geldermann H (2003). Linkage and QTL mapping for Sus scrofa chromosome 12. J Anim Breed Genet.

